# Clinical Improvements Following a Non-Aerobic Therapeutic Exercise in Women with Long COVID

**DOI:** 10.3390/jcm14248786

**Published:** 2025-12-11

**Authors:** María Miana, César Moreta-Fuentes, Ricardo Moreta-Fuentes, David Varillas-Delgado, Carmen Jiménez-Antona, Sofía Laguarta-Val

**Affiliations:** 1 Faculty of Nursing and Physiotherapy Salus Infirmorum, Pontifical University of Salamanca, 28015 Madrid, Spain; mmianaor@upsa.es; 2 Fisyos Center, Método Moreta, 28027 Madrid, Spain; mofuce@gmail.com (C.M.-F.); metodomoreta@gmail.com (R.M.-F.); 3 FREMAP Care Center, 28946 Madrid, Spain; 4 Department of Exercise and Sport Science, Faculty of Health Sciences, Universidad Francisco de Vitoria, 28223 Madrid, Spain; 5 Department of Physical Therapy, Occupational Therapy, Rehabilitation and Physical Medicine, Faculty of Health Sciences, Universidad Rey Juan Carlos (URJC), 28922 Madrid, Spain; carmen.jimenez@urjc.es (C.J.-A.);; 6 Cognitive Neuroscience, Pain, and Rehabilitation Research Group (NECODOR), Faculty of Health Sciences, Rey Juan Carlos University, 28922 Madrid, Spain

**Keywords:** Long COVID, exercise therapy, body composition, fatigue, quality of life

## Abstract

**Background/Objectives**: Long COVID (LC) is characterized by persistent symptoms such as fatigue, pain, and reduced quality of life, often lasting months after acute infection. Exercise-based interventions have shown promise, but evidence for non-aerobic programs remains limited. This study aimed to evaluate the effects of a 12-week motor control exercise program on body composition and fatigue in women with LC and to explore associations with physical activity and psychosocial factors. **Methods**: An exploratory pre–post non-controlled intervention study was conducted in 17 women with LC symptoms persisting for over one year. Participants completed 24 individualized sessions of a non-aerobic therapeutic exercise program focused on trunk stabilization. Outcomes included body composition (bioimpedance analysis), fatigue (Modified Fatigue Impact Scale), health-related quality of life (EQ-5D-5L), physical activity (IPAQ), and kinesiophobia (TSK-11). Paired *t*-tests, effect sizes, correlations, and regression models were applied. **Results**: The intervention significantly reduced total body fat (37.09% to 35.41%, *p* < 0.001) and trunk fat (35.82% to 33.82%, *p* < 0.001), with large effect sizes. Physical and psychosocial fatigue improved markedly (MFIS physical: 29.71 to 21.06, *p* < 0.001; psychosocial: 6.00 to 4.29, *p* = 0.001), while cognitive fatigue showed non-significant change. Pain/discomfort scores decreased substantially (2.86 to 1.79, *p* < 0.001). Vigorous activity and walking time increased, and sedentary time decreased. No significant changes were observed in muscle mass or kinesiophobia. **Conclusions**: A structured, non-aerobic exercise program can effectively reduce body fat, alleviate fatigue, and improve pain perception in women with LC, supporting its role in rehabilitation. Multimodal strategies may be required to address cognitive symptoms and fear of movement.

## 1. Introduction

Post-acute sequelae of SARS-CoV-2 infection (PASC), commonly referred to as Long COVID (LC), is a multifaceted condition that may involve dysfunction across several organ systems, including but not limited to the respiratory, cardiovascular, neurological, gastrointestinal, and musculoskeletal systems [1]. Clinical manifestations are diverse and frequently include persistent fatigue, dyspnea, cognitive deficits, sleep disturbances, myalgia, headaches, impaired concentration, and psychological symptoms such as anxiety and post-traumatic stress [2,3].

Fatigue is among the most prevalent and debilitating symptoms of LC, often persisting for months and significantly impairing daily functioning [4,5]. It has been consistently associated with reduced quality of life across multiple domains [6]. Symptoms may emerge following recovery from the acute phase of COVID-19, persist from the onset, or fluctuate over time, with periods of remission and recurrence [7]. In LC, fatigue and cognitive symptoms frequently coexist and are tightly interrelated, contributing to multidimensional impairment. More than 80% of patients report “brain fog”—a subjective experience of diminished memory, attention, and processing speed—alongside persistent fatigue [8]. Fatigue is highly prevalent and strongly associated with reduced quality of life in LC [9,10,11]. Moreover, women account for approximately 70–73% of Long COVID patients and exhibit around a 30% higher risk compared to men, making this population particularly relevant for targeted intervention studies [12]. In previously hospitalized patients, recovery from post-COVID fatigue may be protracted, potentially extending over several years [13]. The pathophysiology of LC-related fatigue is multifactorial, involving persistent immune activation, chronic inflammation, central nervous system involvement, and mitochondrial dysfunction [14]. Risk factors include older age, female sex, severity and duration of the acute illness, pre-existing autoimmune conditions, depression, and comorbidities [15,16,17].

Also, LC has a profound and enduring impact on patients’ quality of life (QoL), even in those who report partial recovery. Individuals with LC frequently experience impairments in physical, emotional, and social functioning, as well as persistent fatigue, pain, and cognitive decline. LC significantly impairs quality of life across physical and mental domains [18,19]. Another population-based analysis revealed that LC was associated with a 3–4% reduction in EuroQol 5-dimension (EQ-5D) utility scores (approximately 0.03–0.04 on a 0–1 scale) and a 26% increase in mental disability scores [20]. These symptoms correlate with self-reported cognitive symptoms and diminished psychophysical performance, reinforcing the need for long-term monitoring and rehabilitation strategies.

Beyond LC, non-aerobic exercise modalities have demonstrated benefits in other medically unexplained fatigue conditions, such as chronic fatigue syndrome (CFS) and fibromyalgia. Non-aerobic exercise modalities have shown benefits in conditions like chronic fatigue syndrome and fibromyalgia, suggesting potential for LC rehabilitation [21]. Similarly, in fibromyalgia, randomized controlled trials indicate that tai chi may be superior to aerobic exercise in improving symptom severity and quality of life, with sustained benefits over time [22,23]. Emerging evidence also suggests that these approaches could be effective for Long COVID rehabilitation, with preliminary studies reporting improvements in fatigue, pain, and overall health-related quality of life following tai chi or qigong interventions [24]. These findings underscore that various forms of non-aerobic exercise may offer therapeutic value, and future research should compare different modalities to determine whether specific approaches confer unique advantages.

Aerobic exercise improves LC symptoms but may not be tolerated by all patients, highlighting the need for non-aerobic alternatives [25,26]. However, these approaches may not be suitable for all patients, particularly those experiencing post-exertional malaise or severe fatigue. Non-aerobic interventions, including resistance training, core stabilization, and mind–body practices like tai chi and qigong, have shown promising results in related conditions such as chronic fatigue syndrome and fibromyalgia, and preliminary studies in LC suggest benefits for fatigue, pain, and quality of life [27,28]. Comparative evidence remains limited, underscoring the need to evaluate whether non-aerobic strategies can offer similar or superior outcomes while minimizing symptom exacerbation. Evidence from randomized controlled trials indicates that tailored exercise programs can lead to short-term improvements in fatigue, dyspnea, physical function, and the physical domain of QoL [29,30]. However, tolerance varies considerably, and a substantial proportion of LC patients—particularly those experiencing post-exertional malaise—may not tolerate aerobic exercise [31]. Therefore, individualized prescription and careful monitoring are essential to avoid symptom exacerbation.

Reducing fat mass may help mitigate inflammation and improve outcomes in LC. Emerging evidence suggests that excess adiposity, especially abdominal fat, is associated with persistent symptoms and systemic inflammation in LC patients [32]. Visceral adipose tissue is metabolically active and contributes to a pro-inflammatory milieu through cytokine release, which may exacerbate fatigue, pain, and other LC manifestations [33,34]. Therefore, interventions that reduce fat mass could have therapeutic relevance beyond aesthetic or metabolic considerations, potentially mitigating chronic inflammation and improving functional outcomes.

Therefore, the aims of this study were to assess the effects of a 12-week, 24-session motor control exercise program on fat mass percentage and fatigue in individuals with LC and to explore the relationship between changes in these outcomes and baseline variables such as quality of life, physical activity level, and kinesiophobia. Additionally, the study examined the moderating role of age, weight, and baseline characteristics on the magnitude of observed changes.

## 2. Materials and Methods

### 2.1. Design

An exploratory pre–post non-controlled intervention study was conducted involving women diagnosed with LC, aiming to evaluate the impact of a core-focused plank exercise regimen on body composition and perceived fatigue and to explore associations with physical activity and psychosocial factors.

### 2.2. Participants

Recruitment was conducted from September to November 2022 at the Faculty of Health Sciences Clinic, Rey Juan Carlos University. Individuals affiliated with the Madrid Long COVID Association (AMACOP) expressed interest by contacting a dedicated email address managed by the principal investigator, a full professor at Rey Juan Carlos University. Eligible candidates, identified through consecutive non-probability sampling, received detailed study information and documentation via email and were subsequently invited by phone to attend a screening appointment with the Rehabilitation Physician—an attending doctor at Ramón y Cajal University Hospital and part-time faculty member at Rey Juan Carlos University. All individuals contacted for participation agreed to enroll in the study, resulting in an initial sample of 17 women. Compliance was 100%, and no participants dropped out during the 12-week intervention period. Demographic characteristics at baseline are shown in Table 1.

Inclusion criteria required participants to (i) present symptoms consistent with LC for over one year; (ii) be aged 18 years or older; and (iii) have completed the full COVID-19 vaccination schedule as defined by the Spanish Ministry of Health. Exclusion criteria included: (i) severe cardiovascular disease, (ii) abdominal hernia, (iii) pregnancy, (iv) recent musculoskeletal injury or surgery (within the past year), and (v) the presence of neuromuscular disorders.

All participants provided written informed consent. The study protocol was approved by the Ethics Committee of the Hospital Universitario Fundación Alcorcón (approval code: 21/173) and the confidentiality of the participants was ensured, complying with the Declaration of Helsinki 1964 (latest update 2013).

### 2.3. Assessment Procedures

All evaluations were conducted at the Faculty of Health Sciences, Rey Juan Carlos University (Spain). Segmental body composition was measured using bioimpedance analysis (Tanita BC-545N, Tokyo, Japan) before and after the intervention. To ensure measurement reliability, participants were instructed to avoid vigorous physical activity 24 h prior and to urinate before the assessment.

Baseline data collected included age, weight, height, duration of symptoms, admission, pneumonia, emergency, reinfections, and comorbidities. Body mass index (BMI) was calculated as weight (kg) divided by height squared (m^2^) and categorized according to WHO guidelines [35].

Fatigue was assessed pre- and post-intervention using the Modified Fatigue Impact Scale (MFIS), a validated 21-item instrument commonly used in LC research [28,36]. It evaluates fatigue over the previous four weeks across physical, cognitive, and psychosocial domains, with a total score ranging from 0 to 84. A score ≥ 38 is typically considered indicative of clinically relevant fatigue [37].

Quality of life was assessed by using the EuroQol-5D-5L (EQ-5D-5L) pre- and post-intervention and is a standardized instrument for measuring health-related quality of life, comprising a descriptive system with five dimensions—mobility, self-care, usual activities, pain/discomfort, and anxiety/depression [38]. This instrument allows for the classification of 3125 unique health states, where the state coded as 11,111 represents full health (the best possible state), and 55,555 indicates the worst health state [39].

Self-reported physical activity (PA) was evaluated using the International Physical Activity Questionnaire (IPAQ) at pre- and post-intervention. The IPAQ captured the number of days per week participants engaged in vigorous activity, moderate activity, and walking. Additionally, the typical duration per day spent on each activity was recorded, with activities reported in bouts of at least 10 min [40,41].

The Tampa Scale of Kinesiophobia (TSK-11) was administered at both pre- and post-intervention time points to assess fear of movement and (re)injury. The TSK-11 is a shortened version of the original 17-item instrument, excluding items 4, 8, 9, 12, 14, and 16 [42], Like the full version, the TSK-11 uses a 4-point Likert scale ranging from 1 (“strongly disagree”) to 4 (“strongly agree”). The total score is calculated by summing the responses across the 11 items, yielding a possible range from 11 to 44 points. Higher scores reflect greater levels of kinesiophobia, indicating a stronger fear of movement or reinjury [42,43]. Interpretation of the TSK-11 is based on the total score, where a minimum score of 11 denotes negligible or absent kinesiophobia, and a maximum score of 44 indicates a severe fear of movement due to anticipated pain or injury [43]. This scoring framework allows for the quantification of psychological barriers to physical activity, which may influence rehabilitation outcomes and adherence to exercise-based interventions.

### 2.4. Intervention

Participants engaged in a therapeutic exercise program designed to promote correct body alignment and optimal biomechanics, while minimizing compensatory movements (MORETA program) as previously showed [44]. Throughout the intervention period, all participants maintained their habitual dietary patterns. The program consisted of two 60 min sessions per week, conducted on non-consecutive days, totaling 24 sessions. The intervention focused on trunk stabilization through plank-based exercises, aiming to enhance muscular strength and neuromuscular control of the core musculature [45].

Each session was structured into three phases: a 10 min warm-up, a 40 min core training segment, and a 10 min cool-down. The warm-up included 3 min of specific exercises (e.g., wall sits), 1 min of anterior plank on elbows and feet, 50 sit-ups, and 1 min of sustained sit-ups. The core training phase focused on trunk musculature and incorporated exercises targeting the abdominal muscles, gluteus maximus and medius, pelvic bridge, and various plank positions (anterior plank on elbows or hands with extended arms, and side plank). The cool-down phase consisted of breathing exercises, stretching, and muscle relaxation techniques.

Participants progressively increased their workload throughout the intervention. In the initial sessions, they performed between 150 and 200 sit-ups per class, reaching 300 to 400 repetitions by the end of the program. Each week, more complex exercises were introduced, combining leg or arm movements with abdominal exercises to enhance muscle tone, proprioception, and the acquisition of new motor patterns involved in trunk stabilization.

Progressive workload was applied throughout the intervention. In the pelvic bridge exercise, participants increased their maintenance time from 5 to 9 min, while alternating with sets ranging from 10 to 25 repetitions. For gluteus medius activation, maintenance time progressed from 2 to 4 min, and repetitions increased from 20 to 60 per side, all while maintaining correct postural alignment. During plank exercises, participants extended their hold duration from 15–30 s to up to 60 s.

Gradually, more complex exercises were introduced, incorporating external loads (not exceeding 1 kg) and the use of a fit ball to increase exercise difficulty. These modifications aimed to enhance muscle tone, proprioception, and the involuntary activation of trunk-stabilizing musculature.

Depending on the session’s focus, exercises targeting the trunk and gluteal muscles were alternated with routines emphasizing hip and lower limb strength. For instance, quadriceps split exercises progressed from 10 to 50 repetitions. Similarly, shoulder girdle training evolved from 10 plank push-ups (performed on knees and hands) to 50 repetitions executed on feet and hands, while maintaining all biomechanical parameters required for proper execution. In these exercises, correct activation of the serratus anterior and latissimus dorsi was essential to prevent scapular movement or detachment from the thoracic wall.

Exercise intensity was maintained at a moderately high level throughout the program, as assessed using the Perceived Exertion Rating Category Scale [46].

Given the heterogeneous clinical presentation of LC, the exercise program was fully individualized, considering each participant’s symptoms and baseline characteristics at the start of the intervention. The individualized approach was guided by symptom severity and tolerance reported at baseline and during each session. For example, participants presenting higher levels of fatigue or musculoskeletal pain performed shorter plank holds and fewer repetitions, while those with better tolerance progressed to more complex exercises, such as plank combined with limb movements. Adjustments were made to ensure safety and adherence, and no participant experienced adverse effects or required discontinuation of the program.

### 2.5. Statistical Analysis

Analyses were performed using IBM SPSS version 29.0 software (IBM Corp, Armonk, NY, USA). Baseline characteristics (age, weight, and other initial variables) were summarized using means and standard deviations for continuous variables, and frequencies and percentages for categorical variables.

Given the quasi-experimental design with pre–post measurements in a single group of women with LC, the following statistical procedures were applied: The Shapiro–Wilk test was used to assess the normality of the distribution of continuous variables.

To evaluate changes in body composition, MFIS, EQ-5D-5L, IPAQ and TSK-11 pre- and post-intervention paired *t*-tests were used. Cohen’s D effect size (ES) was reported for paired comparisons to quantify the magnitude of the intervention effects. Given the number of paired comparisons and correlation analyses, significance levels were adjusted for multiple testing using the Benjamini–Hochberg False Discovery Rate (FDR) procedure. For transparency, original and corrected *p*-values are reported. Additionally, all *t*-tests include the t-statistics and degrees of freedom.

To assess the influence of age, weight, and baseline characteristics on the magnitude of change in the dependent variables, multiple linear regression models were constructed. Change scores (post–pre) in fat mass and fatigue served as dependent variables, while covariates were entered as predictors.

A significant level of *p* < 0.05 was set for all statistical tests.

## 3. Results

A total of 17 women presenting symptoms of LC completed the study, with no attrition observed during the intervention period.

### 3.1. Body Composition

The intervention produced significant reductions in body fat across all measured regions (*p* = 0.003), with large ES observed in the trunk (*p* = 0.002, ES = 1.186), right arm (*p* = 0.003, ES = 1.076), and left arm (*p* = 0.031, ES = 0.974). However, fat reduction was not evident in the lower limbs, both in the right leg (*p* = 0.089, ES = 0.574) and left leg (*p* = 0.069, ES = 0.617). Additionally, body water percentage increased significantly from 46.08% to 47.02% (*p* = 0.011, ES = −1.524), suggesting improved hydration status or a shift in body composition favoring lean tissue (Table 2).

In contrast, changes in muscle mass were not statistically significant. Although slight increases were observed in total body muscle (from 39.74% to 40.11%) and in specific regions such as the arms, legs, and trunk, none of these differences reached significance (all *p* > 0.05). These findings indicate that the intervention primarily influenced fat reduction and water balance, with minimal impact on muscle hypertrophy during the study period (Table 2).

### 3.2. Modified Fatigue Impact Scale (MFIS)

The intervention led to a significant reduction in perceived fatigue across multiple domains. The physical sub-scale showed the most pronounced improvement, with scores decreasing from 29.71 to 21.06, corresponding to a large effect size (*p* = 0.001, ES = 1.199). Psychosocial fatigue also declined significantly, from 6.00 to 4.29 (*p* = 0.015, ES = 0.970). Overall fatigue scores dropped from 66.59 to 52.59, reflecting a substantial improvement (*p* = 0.012, ES = 0.936).

Although the cognitive sub-scale showed a reduction from 30.88 to 27.24, this change did not reach statistical significance (*p* = 0.142, ES = 0.465). These findings suggest that the intervention was particularly effective in alleviating physical and psychosocial aspects of fatigue, while cognitive fatigue may require additional or targeted strategies to achieve meaningful improvements (Table 3).

### 3.3. EuroQol-5D-5L (EQ-5D-5L)

Following the intervention, changes in health-related quality of life were assessed using the EuroQoL-5D-5L instrument. Among the five dimensions evaluated, the most notable improvement was observed in the domain of pain/discomfort, which showed a substantial reduction in mean score from 2.86 to 1.79, with a large effect size (ES = 1.293) and a statistically significant result (*p* = 0.001). This suggests a clinically meaningful alleviation of pain symptoms post-intervention. Self-care also demonstrated a moderate improvement (*p* = 0.107, ES = 0.564), with scores decreasing from 1.57 to 1.21. The remaining dimensions—mobility, usual activities, and anxiety/depression—showed small effect sizes and non-significant changes (all *p* > 0.05), indicating limited impact of the intervention on these aspects of daily functioning and mental health.

In addition to the dimensional analysis, the total EQ-5D-5L score, likely reflecting a composite or utility-based index, decreased slightly from 24,030.86 to 22,934.21, with no statistical significance and a small effect size (*p* = 0.725, ES = 0.148). This suggests that while specific domains such as pain/discomfort improved markedly, the overall health status as captured by the EQ-5D-5L remained relatively stable. These findings highlight the sensitivity of the pain/discomfort dimension to the intervention and underscore the importance of domain-specific analysis when interpreting EQ-5D-5L outcomes (Table 4).

### 3.4. International Physical Activity Questionnaire (IPAQ)

Following the intervention, several dimensions of physical activity assessed by the IPAQ showed meaningful changes. Notably, vigorous physical activity (IPAQ-1) significantly increased from 0.77 to 1.85 days per week (*p* = 0.048, ES = −0.698), indicating a substantial improvement in high-intensity exercise engagement. Similarly, time spent walking (IPAQ-4.2) rose markedly from 27.50 to 63.75 min per day (*p* = 0.041, ES = −1.415), and the number of days walking at least 10 min (IPAQ-4.1) decreased significantly from 1.80 to 1.03 (*p* = 0.020, ES = 1.789). Additionally, time spent sitting on weekdays (IPAQ-6) decreased significantly from 1.57 to 1.13 h (*p* = 0.036, ES = 1.069), suggesting a reduction in sedentary behavior.

Other IPAQ items did not show statistically significant changes. Moderate physical activity (IPAQ-2) slightly decreased, while walking frequency (IPAQ-3), time spent sitting on weekends (IPAQ-5), and total physical activity time (IPAQ-7) showed minor, non-significant variations (all *p* > 0.05). These results suggest that the intervention was particularly effective in increasing vigorous activity and walking behavior, while its impact on moderate activity and overall sedentary time was more limited (Table 5).

### 3.5. Tampa Scale of Kinesiophobia (TSK-11)

Changes in kinesiophobia were evaluated using the TSK-11. The total score showed a slight reduction from 24.29 pre-intervention to 23.29 post-intervention, with no statistically significant difference and a small effect size (*p* = 0.863, ES = 0.172). This suggests that the intervention had a limited impact on overall fear of movement or reinjury. Among individual items, the greatest improvements were observed in the statements “I’m afraid that I might injure myself if I exercise” and “Pain always means I have injured my body,” with moderate effect sizes although neither reached statistical significance (*p* = 0.112, ES = 0.473 and *p* = 0.219, ES = 0.387, respectively). These trends may indicate a partial shift in pain-related beliefs and attitudes toward physical activity.

Conversely, some items showed minimal or even negative changes, such as “My body is telling me I have something dangerously wrong” and “My accident has put my body at risk for the rest of my life,” with negligible effect sizes and non-significant *p*-values. Interestingly, the item “I wouldn’t have this much pain if there weren’t something potentially dangerous going on in my body” increased slightly post-intervention, suggesting a potential reinforcement of maladaptive beliefs in some participants. Overall, while the intervention did not significantly reduce kinesiophobia, certain cognitive aspects related to fear of movement showed promising trends that warrant further investigation in larger samples or with more targeted psychological strategies (Table 6).

### 3.6. Correlations and Multiple Regression Analysis

To further explore the relationships between changes in body composition and psychosocial outcomes, Pearson correlation analyses were conducted between fat mass reduction and changes in MFIS, EQ-5D-5L, IPAQ, and TSK-11. No significant correlations were found between fat mass reduction and changes in MFIS (r = −0.84, *p* = 0.765), overall EQ-5D-5L scores (r = −0.121, *p* = 0.694), IPAQ scores (r = −0.241, *p* = 0.532) or total TSK-11 scores (r = 0.014, *p* = 0.961).

To identify predictors of fat mass reduction, a multiple linear regression model was constructed using changes in fat mass as dependent variant and MFIS, EQ-5D-5L, IPAQ, and TSK-11 as independent variables using age, weight, and baseline characteristics as covariables. The model was statistically significant (F = 4.12, *p* = 0.022), explaining 52% of the variance in fat mass change (adjusted R^2^ = 0.52). Among the predictors, physical fatigue improvement (β = −0.41, *p* = 0.014) and increased walking time (β = −0.36, *p* = 0.032) emerged as significant contributors to fat mass reduction. These findings underscore the interplay between physical function and body composition in women with LC and highlight the relevance of targeting fatigue and mobility in rehabilitation programs.

Pearson correlation analyses revealed that changes in total muscle mass were positively associated with improvements in IPAQ walking time (r = 0.753, *p* < 0.001) and EQ-5D-5L (r = 0.643, *p* = 0.003). Participants who reported greater increases in walking time and better health status tended to show more favorable gains in muscle mass following the intervention. In contrast, no significant correlations were observed between muscle mass change and MFIS or TSK-11, suggesting that psychological factors may have a limited direct influence on muscle hypertrophy in this context.

To further explore predictors of muscle mass change, a multiple linear regression model was constructed including MFIS, EQ-5D-5L, IPAQ, and TSK-11 change scores, as well as age, baseline weight, and demographic variables such as hospitalization, pneumonia history, and reinfection status. The model explained a moderate proportion of the variance in muscle mass change, although no individual predictors reached statistical significance (all *p* > 0.05). These findings suggest that while physical activity and perceived health improvements may contribute to muscle mass gains, the effects are likely multifactorial and may require larger sample sizes to detect robust predictive patterns.

## 4. Discussion

The results of this study demonstrate that a non-aerobic therapeutic exercise program focused on motor control and trunk stabilization produced significant improvements in body fat and overall fatigue, including both the physical and psychosocial fatigue subscales. Quality of life improved, especially in the pain/discomfort domain, increasing vigorous activity and daily walking time. These findings reinforce the effectiveness of individualized therapeutic non-aerobic exercise–based interventions as a rehabilitation strategy in women with LC.

Current evidence indicates that exercise-based interventions can improve fatigue levels, physical fitness, and the physical dimension of quality of life in individuals affected by LC, while also demonstrating good tolerability among participants [31]. These findings add to the growing body of evidence suggesting that individualized and progressive physical rehabilitation programs can alleviate functional symptoms in LC without leading to significant adverse events. Recent reviews on the mechanisms and management of LC highlight the marked clinical heterogeneity of the condition [47], thereby justifying individualized exercise prescription based on patient tolerance. Accordingly, it is essential to assess patients’ clinical status carefully, as exercise may be harmful for those with LC who also present with myalgic encephalomyelitis/chronic fatigue syndrome or post-exertional malaise, in whom physical activity should not be used as a treatment.

The therapeutic exercise program may have induced a favorable metabolic effect, as evidenced by the significant decrease in body fat, particularly in the trunk and upper limb regions. This response may be attributable to a sustained elevation in total energy expenditure, facilitated by the engagement of large muscle groups during trunk stabilization exercises, as well as by the increase in spontaneous physical activity reflected in the IPAQ results, especially in the frequency of vigorous activity and daily walking duration. Although the intervention resulted in significant reductions in fat mass, it is important to note that the primary energy substrate during the plank-based, moderately high-intensity exercises was likely carbohydrates rather than fat, given the anaerobic nature of the program and reliance on glycolytic metabolism. The accumulation of abdominal subcutaneous fat has been identified as a significant factor associated with the presence of LC symptoms in non-hospitalized Chinese patients, suggesting a potential link between excess adiposity and the persistence of LC symptoms [48]. Therefore, the reduction in adipose tissue observed in this study may have clinically relevant implications beyond simple body recompositing. Persistent inflammation is thought to be a central mechanism in the pathophysiology of LC [49], with visceral adipose tissue potentially contributing to this sustained inflammatory state via biological pathways involving the release of proinflammatory cytokines [50].

The response pattern, substantial improvement in physical and psychosocial fatigue, is consistent with the characteristics of the intervention, which emphasizes core stability and functional training to improve physical tolerance and motor control. Given the relatively short 12-week duration of the program, it was probably insufficient to elicit detectable adaptations in cognitive domain. The limited cognitive response suggests that the mental components of fatigue in LC may require targeted interventions to produce meaningful changes, as emphasized in recent reviews addressing the complex phenomenon of disease-related fatigue [51]. In this context, a recent systematic review noted promising results for noninvasive brain stimulation, hyperbaric oxygen therapy, and PEA-LUT administration in improving brain fog symptoms in LC [8].

The observed improvements in physical and psychosocial fatigue contrast with the limited changes in cognitive fatigue, which may reflect the complex interplay between fatigue and cognitive dysfunction in LC. Brain fog, a term widely used to describe deficits in attention, memory, and processing speed, is strongly associated with fatigue and has been documented in over 80% of patients with persistent symptoms [8]. Neuroimaging studies have linked brain fog to disrupted connectivity in prefrontal and limbic circuits, while neurophysiological evidence suggests altered cortical excitability and impaired motor-cognitive integration [52,53]. These mechanisms may explain why interventions focused solely on motor control, such as the present program, do not fully address cognitive symptoms. Future rehabilitation strategies should consider multimodal approaches that combine physical training with cognitive and neurophysiological interventions to target both fatigue and brain fog.

The marked improvement observed in the pain/discomfort domain of the EQ-5D-5L suggests a clinically meaningful reduction in perceived pain following the therapeutic exercise program. This outcome carries direct functional relevance, given that persistent pain represents one of the principal determinants of impaired quality of life in individuals with LC [54]. In patients with LC, chronic musculoskeletal pain was mainly generalized and persistent, often involving multiple joints, particularly the knees, shoulders, cervical, and lumbosacral regions. Most participants described continuous dull pain, occasionally sharp, that worsened with activity or fatigue and was relieved by rest [55]. In individuals with LC, chronic musculoskeletal pain has been associated with altered central nociceptive processing, a phenomenon known as central sensitization. Several studies have reported that between 30% and 70% of patients experiencing persistent pain after SARS-CoV-2 infection exhibit symptoms consistent with this mechanism, as assessed using specific instruments such as the Central Sensitization Inventory (CSI) and that approximately 15% show impairments in conditioned pain modulation (CPM) [56,57].

Although pain in the present study was assessed globally through the pain/discomfort domain of the EQ-5D-5L, the results may be consistent with the presence of sensitization processes contributing to discomfort and reduced quality of life in patients with LC. In the present study, non-aerobic therapeutic exercise may promote adaptive modulation of nociceptive pathways, enhance the effectiveness of descending pain control mechanisms and reduce the central hypersensitivity described in LC.

The intervention appeared to improve functional tolerance to exertion and promote healthier daily activity patterns. Increases in walking time and vigorous activity, together with reduced sedentary behavior, suggest a gradual reintegration of movement into daily life. People living with LC are exposed to the detrimental effects of prolonged sedentary behavior and physical inactivity, as many reduce their daily activities either as a consequence of, or in an attempt to avoid, post-exertional malaise (PEM) [58]. Although total muscle mass did not change significantly, its correlation with walking time indicates a trend toward better muscle functionality and more efficient use of existing lean mass.

Despite the strengths, this study has some limitations that should be considered

(i) the pre–post design and the relatively small sample size, composed exclusively of women, limit the generalization of the results and the ability to infer causality, (ii) bioelectrical impedance analysis may not detect subtle changes in muscle mass, and the short follow-up period does not allow conclusions about the long-term persistence of the effects, (iii) this study did not compare the MORETA program with other non-aerobic exercise modalities, such as tai chi or qigong, which have demonstrated efficacy in chronic fatigue syndrome, fibromyalgia, and LC. Therefore, it remains unclear whether the observed benefits are specific to trunk stabilization exercises or reflect a broader effect of non-aerobic activity, (iv) the study did not include a comprehensive assessment of cognitive functions beyond the MFIS cognitive subscale. Given the established relationship between fatigue and cognitive performance in LC, and the prevalence of brain fog, this represents an important limitation. Future studies should incorporate objective neurocognitive measures and explore combined interventions to address both physical and cognitive domains, (v) PEM was not specifically assessed or controlled in this study. Given its potential impact on exercise tolerance and safety, future research should include systematic evaluation of PEM to ensure individualized prescription and minimize symptom exacerbation, and (vi) the consistency observed in the results reinforces the relevance of the findings and supports the need for further research with larger, controlled samples and extended follow-up.

From a clinical perspective, these findings support the integration of individualized, non-aerobic exercise programs into rehabilitation protocols for LC, particularly for patients presenting with persistent fatigue and musculoskeletal pain. The observed improvements in body composition and functional tolerance indicate that structured core-focused interventions can help break the cycle of inactivity and fatigue, facilitating a gradual return to daily activities. Clinicians should consider tailoring exercise intensity and progression to patient tolerance, while monitoring for post-exertional malaise. Given the limited impact on cognitive fatigue and fear of movement, combining physical training with cognitive and psychological strategies may optimize recovery. These findings indicate that individualized, non-aerobic exercise programs may help counteract the cycle of fatigue and inactivity that characterizes LC, facilitating a gradual return to functional autonomy. Future research should include head-to-head comparisons of different non-aerobic interventions to identify potential differential effects and optimize rehabilitation strategies for patients with persistent fatigue syndromes.

## 5. Conclusions

A 12-week non-aerobic therapeutic exercise program significantly reduced body fat and improved physical and psychosocial fatigue in women with LC, while also alleviating pain/discomfort and promoting healthier activity patterns. Cognitive fatigue and kinesiophobia showed limited changes, suggesting that multimodal strategies may be needed to address all dimensions of the condition.

## Figures and Tables

**Table 1:** Demographic characteristics of LC patients.

		LC (*n* = 17)
	Age (years), mean (SD)	44.88 (7.23)
	Weight (kg), mean (SD)	69.19 (13.11)
	Height (m), mean (SD)	1.63 (0.64)
	BMI (kg/m^2^), mean (SD)	26.01 (4.56)
	Days of acute COVID-19 symptoms *, mean (SD)	19.01 (9.79)
Admission	Yes, *n* (%)	3 (17.6)
Pneumonia	Yes, *n* (%)	4 (23.5)
Emergency	Yes, *n* (%)	9 (52.9)
COVID Reinfection	Yes, *n* (%)	3 (17.6)
Comorbidities	Yes, *n* (%)	10 (58.8)

BMI, body mass index; kg, kilogram; m, meter. * This refers to the duration of acute symptoms during the initial infection, not LC duration.

**Table 2:** Body composition pre- and post-intervention.

		Pre-Intervention, Mean (SD)	Post-Intervention, Mean (SD)	ES	df	t Value	FDR-Adjusted *p* Value
Fat	Total body (%)	37.09 (7.09)	35.41 (7.47)	1.079	16	4.180	0.003
	Right arm (%)	36.94 (8.58)	35.43 (8.54)	1.076	16	4.169	0.003
	Left arm (%)	37.50 (8.09)	36.14 (8.26)	0.974	16	3.774	0.031
	Right leg (%)	39.41 (5.61)	38.20 (6.91)	0.574	16	2.224	0.089
	Left leg (%)	38.89 (6.13)	37.45 (6.86)	0.617	16	2.389	0.069
	Trunk (%)	35.82 (8.09)	33.82 (8.21)	1.186	16	4.594	0.002
Muscle	Total body (%)	39.74 (3.68)	40.11 (3.19)	−0.223	16	−0.803	0.524
	Right arm (%)	1.92 (0.25)	1.95 (0.22)	−0.277	16	−1.075	0.474
	Left arm (%)	1.99 (0.29)	1.97 (0.24)	0.457	16	0.764	0.553
	Right leg (%)	6.82 (0.67)	6.90 (0.61)	−0.403	16	−0.786	0.542
	Left leg (%)	6.74 (0.63)	6.83 (0.57)	−0.269	16	−1.041	0.445
	Trunk (%)	22.26 (1.92)	22.40 (1.66)	−0.180	16	−0.697	0.603
Body water (%) *		46.08 (4.73)	47.02 (5.08)	−1.524	16	−3.584	0.011

df, degree of freedom; ES, effect size; FDR, False Discovery Rate; SD, standard deviation. * Body water (%) indicates total body water estimated by bioimpedance analysis, independent of fat and muscle percentages.

**Table 3:** Participants’ fatigue pre- and post-intervention measured by Modified Fatigue Impact Scale (MFIS).

	Pre-Intervention, Mean (SD)	Post-Intervention, Mean (SD)	ES	df	t Value	FDR-Adjusted *p* Value
Physical sub-scale	29.71 (4.91)	21.06 (7.64)	1.199	16	4.944	0.001
Cognitive sub-scale	30.88 (4.56)	27.24 (7.12)	0.465	16	1.916	0.142
Phychosocial sub-scale	6.00 (1.73)	4.29 (2.25)	0.970	16	3.998	0.015
Overall fatigue	66.59 (9.26)	52.59 (15.62)	0.936	16	3.861	0.012

df, degree of freedom; ES, effect size; FDR, False Discovery Rate; SD, standard deviation.

**Table 4:** Participants’ quality of life pre- and post-intervention measured by EuroQol-5D-5L (EQ-5D-5L).

	Pre-Intervention, Mean (SD)	Post-Intervention, Mean (SD)	ES	df	t Value	FDR-Adjusted *p* Value
Mobility	2.21 (0.80)	2.14 (0.66)	0.098	16	0.366	0.869
Self-care	1.57 (0.64)	1.21 (0.46)	0.564	16	2.110	0.107
Usual activities	2.86 (0.94)	2.71 (1.04)	0.215	16	0.806	0.546
Pain/discomfort	2.86 (0.66)	1.79 (0.58)	1.293	16	4.836	0.001
Anxiety/depression	2.29 (1.19)	2.07 (0.99)	0.163	16	0.611	0.697
Total	24,030.86 (8307.44)	22,934.21 (6766.54)	0.148	16	0.553	0.725

df, degree of freedom; ES, effect size; FDR, False Discovery Rate; SD, standard deviation.

**Table 5:** Participants’ physical activity pre- and post-intervention measured by International Physical Activity Questionnaire (IPAQ).

	Pre-Intervention, Mean (SD)	Post-Intervention, Mean (SD)	ES	df	t Value	FDR-Adjusted *p* Value
IPAQ-1	0.77 (0.63)	1.85 (1.14)	−0.798	16	−2.452	0.048
IPAQ-2	1.67 (1.15)	1.05 (0.32)	0.277	16	1.000	0.479
IPAQ-3	2.00 (0.88)	2.21 (1.19)	−0.116	16	−0.434	0.796
IPAQ-4.1	1.80 (0.42)	1.03 (0.46)	1.789	16	4.000	0.020
IPAQ-4.2	27.50 (13.23)	63.75 (18.87)	−1.415	16	−2.784	0.041
IPAQ-5	4.88 (2.21)	5.38 (1.62)	−0.254	16	−1.017	0.455
IPAQ-6	1.57 (0.78)	1.13 (0.55)	1.069	16	2.828	0.036
IPAQ-7	7.08 (3.98)	7.92 (2.81)	−0.245	16	−0.847	0.517

df, degree of freedom; ES, effect size; FDR, False Discovery Rate; SD, standard deviation.

**Table 6:** Participants’ kinesiophobia pre- and post-intervention measured Tampa Scale of Kinesiophobia (TSK-11).

	Pre-Intervention, Mean (SD)	Post-Intervention, Mean (SD)	ES	df	t Value	FDR-Adjusted *p* Value
I’m afraid that I might injure myself if I exercise	2.12 (0.87)	1.71 (0.77)	0.473	16	1.951	0.112
If I were to try to overcome it, my pain would increase	2.82 (0.95)	2.41 (0.86)	0.282	16	1.161	0.417
My body is telling me I have something dangerously wrong	2.47 (1.00)	2.53 (0.83)	−0.071	16	−0.293	0.895
People aren’t taking my medical condition seriously enough	2.29 (0.92)	2.00 (0.93)	0.281	16	1.159	0.399
My accident has put my body at risk for the rest of my life	2.12 (0.85)	2.18 (0.93)	−0.054	16	−0.223	0.921
Pain always means I have injured my body	2.18 (0.86)	1.76 (0.83)	0.387	16	1.595	0.219
Simply being careful that I do not make any unnecessary movements is the safest thing I can do to prevent my pain from worsening	2.00 (0.85)	2.06 (0.89)	−0.044	16	−0.180	0.942
I wouldn’t have this much pain if there weren’t something potentially dangerous going on in my body	2.94 (0.89)	3.18 (0.64)	−0.260	16	−1.074	0.477
Pain lets me know when to stop exercising so that I don’t injure myself	1.59 (0.87)	1.53 (0.87)	0.061	16	0.251	0.879
I can’t do all the things normal people do because it’s too easy for me to get injured	2.06 (0.89)	2.00 (0.93)	0.071	16	0.293	0.867
No one should have to exercise when he/she is in pain	1.76 (0.90)	1.94 (0.89)	−0.200	16	−0.824	0.521
Total	24.29 (7.24)	23.29 (5.03)	0.172	16	0.244	0.863

df, degree of freedom; ES, effect size; FDR, False Discovery Rate; SD, standard deviation.

## Data Availability

The data presented in this study are available on request from the corresponding author. The data are not publicly available due to legal restrictions.
